# Primary Care Patients’ Preference for Hospitals over Clinics in Korea

**DOI:** 10.3390/ijerph15061119

**Published:** 2018-05-30

**Authors:** Agnus M. Kim, Seongcheol Cho, Hyun Joo Kim, Hyemin Jung, Min-Woo Jo, Jin Yong Lee, Sang Jun Eun

**Affiliations:** 1 Department of Health Policy and Management, Seoul National University College of Medicine, Seoul 03080, Korea; agnus@snu.ac.kr (A.M.K.); hyeminjung82@gmail.com (H.J.); 2 Regional Emergency Care Center, Internal Medicine, Seoul National University Hospital, Seoul National University College of Medicine, Seoul 03080, Korea; mdchosc@gmail.com; 3 Department of Nursing Science, Shinsung University, Dangjin 31801, Korea; hyjkim2012@gmail.com; 4 Department of Preventive Medicine, University of Ulsan College of Medicine, Seoul 05505, Korea; mdjominwoo@gmail.com; 5 Public Health Medical Service, Boramae Medical Center, Seoul National University College of Medicine, 20 Boramae-ro 5-gil, Dongjak-gu, Seoul 07061, Korea; 6 Department of Preventive Medicine, Chungnam National University College of Medicine, Daejeon 35015, Korea

**Keywords:** primary care, health care facilities, access, preference, hospitals, clinics, Korea

## Abstract

Korea is in a unique condition to observe whether patients, when equal access to the levels of health care facilities is guaranteed by the support of the national health insurance, choose the appropriate levels of health care facilities. This study was performed to investigate the primary care patients’ preference for hospitals over clinics under no restriction for their choice. We used the 2011 National Inpatient Sample database of the Health Insurance Review and Assessment Service in Korea. A primary care patient was defined as a patient who visited as an outpatient in health care facilities with one of the 52 minor conditions defined by the Korean government. We found that approximately 15% of outpatient visits of the patients who were eligible for primary care in Korea happened in hospitals. In terms of cost, the outpatient visits in hospitals accounted for about 29% of total cost of outpatient visits. This arbitrary access to hospitals can lead to an inefficient use of health care resources. In order to ensure that health care facilities are stratified in terms of access as well as size and function, interventions to distribute patients to the appropriate level of care are required.

## 1. Introduction

In many developed countries, the roles of health care facilities are differentiated based on their function and size [1,2,3,4]. The level of a health care facility is chosen according to the severity of the patients’ health conditions. Patients with minor and chronic illnesses are normally treated in primary care settings, while hospitals provide specialized treatments for more complex cases, which could involve hospitalization. This division of roles among health care facilities has been institutionalized through the course of development of the modern health care system and is believed to be fit for an organized and efficient provision of health care services.

In Korea, which boasts of its achievement of universal health coverage in a short period of time, the levels of the health care facilities are not clearly divided [5]. Patients can access any level of health care facilities for their first and ensuing contacts under nominal restrictions which are hardly working. This situation has long been a controversial issue among patients, health care providers, and government in Korea [5]. Patients are claiming their rights to choose the most qualified physician, who, they believe, are in large-sized hospitals, which are usually located in larger metropolitan areas [6]. Physicians in clinics, emphasizing the importance of primary care, which guarantees long and continuous care, criticize hospitals for taking away their potential patients [7]. Hospitals criticize the iniquitously low inpatient fee schedule which compels them to augment the ambulatory care services [8]. The Korean government has attempted to establish the stratified health care delivery system with various measures, such as the forced designation of health service area, referral slip for visiting tertiary hospitals, and differential coinsurance for outpatient and outpatient prescription among the levels of health care facilities, which proved to have little effect [5,9,10,11]. To settle this conflict, the arguments need to be examined in terms of the justifiability and feasibility, which should be weighed against the efficient use of health care resources.

Given that enhancing health coverage is a matter of paramount importance globally, the conflict in Korea concerning access to health care is worthy of a careful investigation. Due to its specificity to the Korean health care context, the issue has mainly been reported in the literature from Korea [5,6,11,12]. However, most of the studies did not elaborate the description in terms of patients’ characteristics. To determine whether the expansion of the outpatient sector in hospitals relates to the overuse of the patients who were originally fit for hospitals or to misuse of the patients who can be treated in clinics, the outpatient care use should be specified in terms of patients’ characteristics. In addition, the prior studies characterized the health care utilization in Korea as being unjustifiable, based on the mandate that the access to health care facilities should be divided and restricted by their level. However, to deepen our understanding of this phenomenon, the study needs to focus on some fundamental principles, such as the patients’ behavior in certain health care circumstances. Korea is in a unique condition to observe whether patients, under the extensive support of a third-party payer, choose the levels of health care facilities which are appropriate to treat their health conditions. As an attempt to examine the pattern of health care utilization, when equal access to the levels of health care facilities is guaranteed by the support of the national health insurance, this study investigated primary care patients’ preference for hospitals over clinics in Korea and analyzed the phenomenon in terms of the recent development of the health care system in Korea.

## 2. Materials and Methods

We used the National Inpatient Sample (NIS) data for the 2011 period, and we obtained Institutional Review Board approval from Chungnam National University School of Medicine (IRB No. 14-05). The NIS is a nationally representative sample of the Health Insurance Review and Assessment Service (HIRA), which covers the claims data of 90% of the total population in Korea: 46 million patients. The NIS is extracted by a stratified randomized sampling method and includes 13% of the total annual inpatient claims and 1% of the annual outpatient claims [13]. The 2011 NIS contains 29,837,213 claims from outpatient services, of which 4,902,210 claims were from conventional medical facilities. Claims of oriental medicine, dentistry, and mental health facilities were excluded. Each claim in the database is designed to represent 100 claims.

We defined a primary care patient as a patient who visited health care facilities as an outpatient with minor medical conditions which could be treated in primary care settings. To specify “minor conditions”, we first adopted the 52 minor conditions selected by the Korean government. In 2011, as a measure to prevent hospital utilization by primary care patients, the Korean government designated the 52 minor conditions, for which prescription fees from hospital outpatient visits would be imposed at a higher coinsurance rate [14]. The 52 minor conditions cover common chronic diseases and acute conditions which can be properly managed in primary care (Table A1). Second, we excluded the outpatient claims of patients with a history of hospital admission in 2011, and the outpatient visits with two or more minor conditions were also excluded. Third, in order to refine our definition of minor conditions which do not require hospital outpatient utilization, we employed the scoring of the Charlson comorbidity index (CCI), a composite score calculated by summating the weighted relative risks of one-year mortality of the 17 comorbid conditions [15]. We determined that a comorbidity score of 0 could corroborate the inappropriateness of the hospital outpatient visits of the patients with a diagnosis among the 52 minor conditions. When a patient’s minor condition was included in the 17 comorbid conditions in the CCI, we calculated a CCI score after excluding the overlapped condition. Through this process, 1,725,985 outpatient claims were finally selected from 4,902,210 claims of conventional health care facilities. As each claim or patient was designed to represent 100 claims in the 2011 NIS database, this sampling weight was applied to all statistical analyses in this study.

We classified the outpatient claims in 2011 with a diagnosis among 52 minor conditions without prior history of hospitalization in the same year and with a CCI score of 0 by health care facilities. In the Korean health care system, the parent category of “hospitals,” includes subcategories of hospitals, general hospitals, and tertiary hospitals, the requirements for whose qualifications are stated by Korean law. As a subcategory, a hospital signifies a small hospital in Korea (30–100 beds). To prevent confusion between the concept of a hospital as an umbrella term and its subcategory, we designated the subcategory of a small hospital. General hospitals are hospitals equipped with more than 100 beds and several specialty departments as designated by law, and tertiary hospitals are large-sized university hospitals selected by the government. As of 2011, 44 university hospitals were designated as tertiary hospitals.

We estimated the total number and cost of claims by the types of health care facilities. The costs were calculated separately by the National Health Insurance coverage and the out-of-pocket costs. All costs are presented in United States dollars (USD), with the year’s average exchange rate of 2011 (1 USD = 1151.8 Korean won). In order to identify the difference in outpatient visits by the types of medical facilities, chi-squared tests for trend were performed. Analysis of variance (ANOVA), a statistical method that assess differences in a continuous variable, was used to identity the differences in annual average healthcare cost by the types of healthcare facilities, and we confirmed the difference among each health care facility by the Tukey’s b test. All the analyses were performed using SAS version 9.2 (SAS Institute, Inc., Cary, NC, USA) and SPSS 20 (IBM Corporation, Armonk, NY, USA). All statistical tests were two-sided, and a *p*-value < 0.05 was considered statistically significant.

## 3. Results

We analyzed the outpatient utilization of primary care patients in Korea by the types of health care facilities, using the estimated number and costs of annual visits. A primary care patient was defined as a patient with a single diagnosis within 52 simple or minor disease groups as defined by the Korean government, without a prior history of hospitalization in the same year, and with a Charlson comorbidity index (CCI) score of 0. The outpatient visits by primary care patients in 2011 are described in Table 1. The total number of outpatient visits by primary care patients was estimated at about 163 million, 15% of which occurred in hospitals, while the rest occurred in clinics. Among the 52 minor conditions, the disease group of gastritis and duodenitis was the most common, taking 23% of total outpatient visits of primary care patients, of which 23% occurred in hospitals. In the case of the second common condition, essential hypertension, 17% of outpatient visits happened in hospitals. Among respiratory diseases, acute bronchitis, acute upper respiratory infections of multiple and unspecified sites, and acute nasopharyngitis were in the top 10 list.

Estimated cost incurred on the National Health Insurance Service (NHIS) and patients are presented in Table A2. The total cost of outpatient visits by primary care patients was estimated at 2642 million USD, of which 28.5% was from hospitals. Of the total cost of outpatient visits, 70% was paid by the NHIS, and 30% was out-of-pocket cost. While the out-of-pocket cost accounted for about 16% of total cost of outpatient visits in clinics, 44% of the total cost of outpatient visits in hospitals was out-of-pocket cost. As in the case of number of claims, gastritis and duodenitis showed the highest cost and essential hypertension showed the second highest.

We compared our results with the outpatient claim records of the entire population in Korea in 2011 issued by the National Health Insurance Corporation (Table 2) [16]. The estimated outpatient claims by primary patients accounted for 27% in the number and 23% in the cost of total outpatient claims in Korea. The proportion of hospital outpatient claims in primary care patients was 15% in number and 29% in cost, both of which were lower than that of the entire population (16% and 43%, respectively). However, the proportion of the number of outpatient claims in small hospitals was higher in primary care patients at 7% than in the entire population at 5%.

## 4. Discussion

In this study, we investigated the primary care patients’ preference for hospitals over clinics under no restriction for their choice. A primary care patient was defined as having a single diagnosis among 52 minor conditions without prior history of hospitalization in the same year and with a CCI score of 0 by health care facilities. Of total outpatient visits by primary care patients in 2011, 15% occurred in hospitals, and their costs accounted for 28.5% of total outpatient visits by primary care patients. The higher proportion of expenditure compared to the number of visits reflects the current outpatient fee schedule, which applies a higher fee to the higher levels of health care facilities. Compared with the outpatient claims of the entire population, the proportion of outpatient claims of primary care patients was higher in small hospitals.

Of the total 163 million outpatient visits by primary care patients in Korea, 15% occurred in hospitals. This proportion is likely to be high compared with other countries. In many developed countries, outpatient hospital visits are usually preceded by visits to clinics, and in some European countries, visits to clinics are mandatory before visiting a hospital [1,2,3,4,11,17]. In the U.S., hospital outpatient visits accounted for about 9% of total outpatient visits [18] and more than half of hospital outpatient visits were referred cases [19]. Therefore, practically, less than 5% of all outpatient visits in the U.S. occur in hospitals without referral. If the outpatient visits are restricted only to primary care patients, who are less likely to require complex treatment, this proportion will further decrease.

The diagnoses in the upper 10 categories show common single reasons for primary care patients’ outpatient visits. The group of gastritis and duodenitis, the 12th biggest cause of outpatient visits of patients in general in Korea [20], was the most common cause for outpatient visits of primary care patients. Acute bronchitis, the most common cause of outpatient visits of patients in general in Korea, was the fourth most common cause of outpatient visits of primary care patients. This status of acute bronchitis, as a main cause of outpatient visits, is unusual compared with other countries [21]. Acute bronchitis, as well as acute upper respiratory infections of multiple and unspecified sites, and acute nasopharyngitis, which were in the top 10 list, are the diagnoses applied for patients with a common cold. In Korea, antibiotic prescription for a common cold is applicable to an arbitrary retrieval of insurance reimbursement, which is considered inappropriate by physicians. A high proportion of acute bronchitis cases is likely to be a result of an upcoding to avoid the retrieval.

The comparison of our results with the outpatient use of patients in general (Table 2) provides a more detailed explanation about the use of health care facilities in Korea. Outpatient hospital services were more frequently used by primary care patients than by patients in general only in small hospitals. This result shows that small hospitals, which make up 80% of all hospitals in number [22], are the typical product of the Korean health care system at the boundary between hospitals and clinics both in terms of their role and size. Tertiary hospitals, which comprise only 3% of all hospitals in number, accounted for 27% of all hospital outpatient expenditure for primary care patients. This proportion, although attenuated compared to in the case of patients in general, which was at 40%, shows the definite preference for higher levels of health care facilities among patients in Korea, which includes patients with minor conditions.

The unique status of hospitals as primary care facilities relates to how health care facilities have been established in Korea. The rapid transition from an absolute lack of health care facilities just decades ago to the recent abundance of health care facilities involved a haphazard increase in health care facilities, which was mainly driven by the private sector [23], and in the course of this rapid transition, many private clinics developed into hospitals [24]. A sharp increase in the number of health care facilities, unaccompanied by clear division of their roles, made the boundaries blurred. Hospitals kept taking care of outpatients with minor conditions as they did in the past when hospitals were only the available health care facilities in the paucity of clinics, and clinics performed inpatient care as commonly practiced in the past. As a result, outpatient expenditure accounts for a third of total expenditure in hospitals, and inpatient expenditure is about 10% of the total expenditure in clinics [5].

As a consequence of private sector-led increase in health care facilities, private ownership has become a prevalent form of ownership of health care facilities in Korea, with 60% of all hospitals being privately-owned [22]. Ownership affects the behavior of health care facilities, and private ownership makes the hospitals more profit-sensitive as profit is essential for sustaining the existence of private hospitals in the market. In the Korean health care system, where the inpatient fee is excessively low, maintaining outpatient services which are more lucrative and performing services whose costs are not covered by insurance and therefore, whose prices are not regulated by government, have been survival strategies for privately-owned hospitals [25,26].

Outpatient hospital care in Korea has been on the rise for the last few decades. This is related to the overall expansion of the hospital sector. While the outpatient expenditure of clinics increased by 80% between 2005 and 2015, the expenditure for outpatient hospital care has increased by 173%, and their proportion of total fees for outpatient care increased by 10% [27]. The total expenditure of clinics and hospitals showed a similar change, with a markedly higher growth in hospital expenditure. This trend shows a stark contrast with other countries, where the proportion of expenditure between hospitals and clinics remains steady [28,29]. In addition, during the same period, the proportion of hospital outpatient care in the total hospital expenditure was stable at around 35%, which is lower than and in contrast with other countries where the proportion of revenue from outpatient services relative to inpatient services has been augmented [30,31,32,33]. These results suggest that the hospitals’ high share of outpatient care in Korea could be a collateral consequence of disproportionate growth of the hospital sector, rather than the result of the expansion of outpatient care within hospitals.

The persistence of the undifferentiated access to health care facilities through the past decades has affected the public perception, which is also reflected in language. Unlike in Western countries, where hospitals and clinics had different origins and their own distinctive roles, in Korea, the term “hospital”, which was introduced in the early 1900s as a modern form of health care facilities, was the only concept designating health care facilities, regardless of their size and function, until the 1970s, when the term “clinic” was designated by the law [34]. Still, despite the frequent use of clinics, the term “hospital” in Korea, as in dictionary and common usage [35], is recognized as a general term covering all the health care facilities for treating patients, rather than as an institution specified in terms of its size and function. In the situation where arbitrary access to health care facilities is taken as a given by patients, changing an already established system can provoke a strong opposition from the public, which the policy makers would be most afraid of. The public perception which has been maintained and reinforced by the interaction of the health care system and culture has made it difficult to take any practical measures that could preclude the arbitrary access to health care facilities. Therefore, a means whereby to change the public perception which has been ingrained in the course of the development of the health care system in Korea would be key in implementing a measure to differentiate the level of health care facilities.

This study has some limitations. First, this study is a cross-sectional study performed with the 2011 NIS data. Therefore, it is difficult to see how the outpatient visits changed with the type of health care facilities. Considering that there were changes in the outpatient cost-sharing policies in Korea in 2009 and 2011 [10,36], a longitudinal study would be useful to assess the effect of those policies. Second, the lack of literature from countries other than Korea made it difficult to assess the degree of the patients’ preference of hospitals over clinics in comparison with other countries. Third, in terms of the analysis, the causes for the patients’ choice of hospitals over clinics were not thoroughly investigated. As mentioned in the introduction, the overuse of hospital outpatient service can be due to the absence of compulsory measures that prevent arbitrary access to health care facilities or little differentiation in the amount of out-of-pocket expenses. However, regardless of the current lack of the regulatory measures, the preference for hospitals among patients in Korea is likely to be internalized through the course of development of the Korean health care system. Our study might serve as a starting point for further investigation of such factors inherent in the Korean population. Despite these limitations, this study has its own value as an elaborate description of how patients behave when they are granted access to all levels of health care facilities with the support of the third-party payer.

In this study, we observed that the preference for higher levels of health care facilities in Korea could be higher than in other countries, and the division of roles among health care facilities was not established even in the case of primary care patients. This phenomenon is partly rooted in the haphazard increase in health care facilities without appropriate measures to divide their roles. The measures such as fee differentiation for outpatient visits and prescriptions, which have been hitherto adopted and have been proven to have little effect [10,36], might have been too weak to curb the patients’ strong preference for the higher levels of health care facilities. These issues should be further investigated in future studies. Unwarranted preference for higher levels of health care facilities is not efficient and effective as it can incur additional costs resulting from more resource use, and possibly, unnecessary interventions. Measures to redress the health care delivery system and public perception should be instituted.

## 5. Conclusions

For the past decades, Korea has gone through a rapid expansion of health care coverage and a sharp increase in health care facilities, which could be achieved under the auspices of the national health insurance. However, this growth was not accompanied by the proper measures to control the unnecessary and inappropriate use of health care services. The support of a strong third-party payer, national health insurance, can relieve the insured of the financial burden and at the same time, deprive the insured of the motivation for rational choice. Our study results concerning patients’ choice of health care facilities provide an example. Patients in Korea would choose higher levels of health care facilities than were needed when they were made equally accessible with the support of the national health insurance. This could lead to the inefficient use of health care resources and, possibly, the loss of benefits to those who could potentially benefit. In order to ensure that health care facilities are stratified in terms of access as well as size and function, interventions to distribute patients to the appropriate levels of care are required.

## Figures and Tables

**Table 1:** Estimated number and percentages of outpatient visits by the types of health care facilities.

	Disease Groups	Total	Clinics	Hospital Total	H ^1^ Small	H General	H Tertiary	*p*-Value
Total ^2^		162,937,477 (100.0)	138,181,262 (84.8)	24,756,214 (15.2)	10,922,051 (6.7)	9,518,043 (5.8)	4,316,121 (2.6)	<0.001
1	Gastritis and duodenitis	37,269,075 (100.0)	28,731,857 (77.1)	8,537,218 (22.9)	4,404,709 (11.8)	3,388,908 (9.1)	743,602 (2.0)	<0.001
2	Essential hypertension	14,928,745 (100.0)	12,427,440 (83.2)	2,501,305 (16.8)	739,802 (5.0)	1,082,102 (7.2)	679,401 (4.6)	<0.001
3	Vasomotor and allergic rhinitis	11,517,229 (100.0)	10,576,420 (91.8)	940,810 (8.2)	457,906 (4.0)	337,303 (2.9)	145,601 (1.3)	<0.001
4	Acute bronchitis	11,404,311 (100.0)	10,619,293 (93.1)	785,017 (6.9)	439,209 (3.9)	289,807 (2.5)	56,001 (0.5)	<0.001
5	Dyspepsia	9,573,130 (100.0)	8,756,828 (91.5)	816,302 (8.5)	527,201 (5.5)	223,000 (2.3)	66,100 (0.7)	<0.001
6	Conjunctivitis	8,920,162 (100.0)	8,513,859 (95.4)	406,303 (4.6)	148,801 (1.7)	182,901 (2.1)	74,601 (0.8)	<0.001
7	Acute upper respiratory infections of multiple and unspecified sites	5,738,277 (100.0)	5,259,171 (91.7)	479,106 (8.3)	270,103 (4.7)	154,102 (2.7)	54,901 (1.0)	<0.001
8	Allergic contact dermatitis	5,327,116 (100.0)	5,101,515 (95.8)	225,601 (4.2)	53,200 (1.0)	96,201 (1.8)	76,200 (1.4)	<0.001
9	Acute nasopharyngitis	4,158,661 (100.0)	3,869,358 (93.0)	289,304 (7.0)	152,902 (3.7)	101,201 (2.4)	35,200 (0.8)	<0.001
10	Disorders of lipoprotein metabolism and other lipidemias	3,991,727 (100.0)	2,404,120 (60.2)	1,587,607 (39.8)	213,001 (5.3)	651,802 (16.3)	722,804 (18.1)	<0.001

^1^ H: Hospitals. ^2^ Sum of the estimated numbers of visits among 52 minor conditions.

**Table 2:** Number and claim costs of outpatient visits in primary care patients and patients in general.

		Total	Clinic	Hospitals Subtotal	H ^1^ Small	H General	H Tertiary
No. of claims	Patients in general	605,084,745 (100)	509,004,867 (84)	96,079,878 (16)	32,959,412 (5)	38,109,031 (6)	25,011,435 (4)
	Primary care patients	162,937,477 (100)	138,181,262 (85)	24,756,215 (15)	10,922,051 (7)	9,518,043 (6)	4,316,121 (3)
Costs of claims	Patients in general	13,294 (100)	7540 (57)	5754 (43)	1292 (10)	2139 (16)	2323 (17)
	Primary care patients	3044 (100)	2175 (71)	869 (29)	281 (9)	358 (12)	231 (8)

^1^ H: Hospitals.

## Appendix A

**Table A1:** The list of 52 simple or minor disease groups as designated by the Ministry of Health and Welfare in Korea.

	Disease Groups	ICD^1^-10 Codes
1	Other gastroenteritis and colitis of infectious and unspecified origin	A09.0–09.9
2	Dermatophytosis	B35.2–B35.6, B35.8, B35.9
3	Non-insulin-dependent diabetes mellitus	E11.2–E11.9
4	Disorders of lipoprotein metabolism and other lipidemias	E78.0–E78.9
5	Hordeolum and chalazion	H00.0, H00.1
6	Disorders of lacrimal system	H04.0–H04.9
7	Conjunctivitis	H10.0–H10.9
8	Senile cataract	H25.0–H25.9
9	Disorders of refraction and accommodation	H52.0–H52.7
10	Otitis externa	H60.1, H60.3, H60.5, H60.8, H60.9
11	Essential hypertension	I10.0, I10.9
12	Acute nasopharyngitis	J00
13	Acute sinusitis	J01.0–J01.9
14	Acute pharyngitis	J02.0–J02.9
15	Acute tonsillitis	J03.0–J03.9
16	Acute laryngitis and tracheitis	J04.0–J04.2
17	Acute upper respiratory infections of multiple and unspecified sites	J06.0–J06.9
18	Acute bronchitis	J20.9
19	Vasomotor and allergic rhinitis	J30.0–J30.4
20	Chronic nasopharyngitis and pharyngitis	J31.1, J31.2
21	Chronic sinusitis	J32.0–J32.9
22	Asthma	J45.0–J45.9
23	Gastro-oesophageal reflux disease	K21.0–K21.9
24	Gastric ulcer	K25.3, K25.7, K25.9
25	Peptic ulcer, site unspecified	K27.3, K27.7, K27.9
26	Gastritis and duodenitis	K29.0–K29.9
27	Dyspepsia	K30
28	Other noninfective gastroenteritis and colitis	K52.2, K52.3, K52.8, K52.9
29	Irritable bowel syndrome	K58.0–K58.9
30	Other functional intestinal disorders	K59.0–K59.2, K59.4, K59.8, K59.9
31	Other diseases of liver	K76.0, K76.9
32	Atopic dermatitis	L20.8, L20.9
33	Allergic contact dermatitis	L23.8, L23.9
34	Urticaria	L50.0–L50.9
35	Other arthritis	M13.0–M13.9
36	Spondylosis	M47.8, M47.9
37	Cervical disc disorders	M50.9
38	Other intervertebral disc disorders	M51.3, M51.4, M51.8, M51.9
39	Dorsalgia	M54.8, M54.9
40	Synovitis and tenosynovitis	M65.2, M65.3, M65.8, M65.9
41	Shoulder lesions	M75.0, M75.2, M75.9
42	Other enthesopathies	M77.8, M77.9
43	Other soft tissue disorders, not elsewhere classified	M79.1, M79.4, M79.6, M79.8, M79.9
44	Osteoporosis without pathological fracture	M81.0–M81.9
45	Cystitis	N30.0, N30.9
46	Chronic prostatitis	N41.1
47	Other inflammation of vagina and vulva	N76.0, N76.2
48	Menopausal and other perimenopausal disorders	N95.1, N95.2, N95.9
49	Sprain and strain of joints and ligaments of lumbar spine and pelvis	S33.5–S33.7
50	Sprain and strain of joints and ligaments at wrist and hand level	S63.6, S63.7
51	Sprain and strain of other and unspecified parts of knee	S83.6
52	Sprain and strain of joints and ligaments at ankle and foot level	S93.5, S93.6

^1^ ICD: International Classification of Diseases.

**Table A2:** The estimated cost due to outpatient visits by the type of health care facilities (Unit: 1,000,000 USD).

Rank	Disease Groups	Total			Clinic			Hospitals Subtotal			H ^1^ Small			H General			H Tertiary			*p*-Value
		Total Cost	NHIS ^3^	OOP ^4^	Total Cost	NHIS	OOP	Total Cost	NHIS	OOP	Total Cost	NHIS	OOP	Total Cost	NHIS	OOP	Total Cost	NHIS	OOP	
Total ^2^		2642	1838	805	1888	1411	477	754	427	328	244	154	90	311	177	134	200	96	104	<0.001
		(100.0)	(69.5)	(30.5)	(71.5)	(53.4)	(18.1)	(28.5)	(16.1)	(12.4)	(9.2)	(5.8)	(3.4)	(11.8)	(6.7)	(5.1)	(7.6)	(3.6)	(3.9)	
1	Gastritis and duodenitis	753	515	238	465	346	119	288	169	119	114	71	43	128	72	56	45	26	20	<0.001
		(100.0)	(68.4)	(31.6)	(61.8)	(46.0)	(15.8)	(38.2)	(22.5)	(15.8)	(15.1)	(9.4)	(5.7)	(17.1)	(9.6)	(7.5)	(6.0)	(3.4)	(2.6)	
2	Essential hypertension	191	135	55	135	105	30	55	30	25	10	6	3	22	13	10	23	11	12	<0.001
		(100.0)	(70.9)	(29.1)	(71.0)	(55.1)	(15.9)	(29.0)	(15.8)	(13.1)	(5.2)	(3.4)	(1.8)	(11.6)	(6.6)	(5.1)	(12.2)	(5.9)	(6.2)	
3	Dyspepsia	173	124	49	148	109	39	25	15	10	13	8	5	8	5	4	3	2	2	<0.001
		(100.0)	(71.5)	(28.5)	(85.5)	(63.0)	(22.5)	(14.5)	(8.5)	(6.0)	(7.7)	(4.8)	(2.9)	(4.8)	(2.7)	(2.1)	(2.0)	(1.0)	(1.0)	
4	Vasomotor and allergic rhinitis	135	96	39	116	86	30	19	10	9	7	4	2	8	4	3	5	2	3	<0.001
		(100.0)	(71.1)	(28.9)	(85.8)	(63.5)	(22.2)	(14.2)	(7.6)	(6.7)	(4.9)	(3.2)	(1.7)	(5.6)	(3.1)	(2.4)	(3.8)	(1.3)	(2.5)	
5	Conjunctivitis	128	95	34	118	89	29	10	5	5	3	2	1	5	3	2	2	1	2	<0.001
		(100.0)	(73.7)	(26.3)	(92.0)	(69.5)	(22.5)	(8.0)	(4.2)	(3.8)	(2.0)	(1.3)	(0.8)	(4.1)	(2.3)	(1.8)	(1.8)	(0.6)	(1.2)	
6	Acute bronchitis	125	93	32	111	84	27	14	8	6	6	4	2	6	4	2	2	1	1	<0.001
		(100.0)	(74.3)	(25.7)	(88.9)	(67.6)	(21.3)	(11.1)	(6.7)	(4.4)	(4.8)	(3.2)	(1.6)	(4.7)	(2.9)	(1.8)	(1.6)	(0.6)	(1.0)	
7	Disorders of lipoprotein metabolism and other lipidemias	114	71	43	45	34	11	69	37	32	5	3	2	24	13	10	40	20	20	<0.001
		(100.0)	(62.3)	(37.7)	(39.4)	(30.0)	(9.5)	(60.6)	(32.3)	(28.2)	(4.5)	(2.8)	(1.7)	(20.7)	(11.6)	(9.0)	(35.4)	(17.9)	(17.5)	
8	Disorders of refraction and accommodation	81	56	26	63	46	17	18	10	9	10	6	4	4	2	2	4	1	3	<0.001
		(100.0)	(68.3)	(31.7)	(77.7)	(56.3)	(21.3)	(22.3)	(11.9)	(10.4)	(11.9)	(7.4)	(4.5)	(5.4)	(3.0)	(2.4)	(5.1)	(1.6)	(3.5)	
9	Other functional intestinal disorders	69	48	21	37	28	9	32	19	12	9	6	3	16	10	7	7	4	3	<0.001
		(100.0)	(69.5)	(30.5)	(53.6)	(40.8)	(12.8)	(46.4)	(28.7)	(17.7)	(13.3)	(8.8)	(4.4)	(23.3)	(13.8)	(9.5)	(9.9)	(6.1)	(3.8)	
10	Acute upper respiratory infections of multiple and unspecified sites	68	49	18	56	42	14	11	7	4	4	2	1	5	3	2	3	1	2	<0.001
		(100.0)	(73.0)	(27.0)	(83.2)	(62.6)	(20.5)	(16.8)	(10.3)	(6.5)	(5.5)	(3.6)	(1.9)	(7.4)	(5.1)	(2.2)	(4.0)	(1.6)	(2.4)	

^1^ H: Hospitals. ^2^ Sum of the estimated cost of visits among 52 minor conditions. ^3^ NHIS: National Health Insurance Service. ^4^ OOP: Out-of-pocket.
